# Kartagener’s syndrome

**DOI:** 10.11604/pamj.2018.29.160.14927

**Published:** 2018-03-19

**Authors:** Rodolfo Mendes Queiroz, Fred Bernardes Filho

**Affiliations:** 1 Department of Radiology and Imaging, Santa Casa da Misericórdia of Avaré, Avaré, São Paulo, Brazil; 2 CENTROMED Diagnóstico por Imagem, Avaré, São Paulo, Brazil; 3 Dermatology Division, Department of Medical Clinics, Ribeirão Preto Medical School, University of São Paulo, Ribeirão Preto, Brazil

**Keywords:** Kartagener´s syndrome, dextrocardia, sinusitis

## Image in medicine

A 29-year-old non-smoker male with a history of chronic cough, and recurrent pneumonia, sinusitis and otomastoiditis was admitted to the emergency room with a 3-day history of headache, cough productive and dyspnea. Positive findings on physical examination included heart sounds in the right side of his chest and pain on palpation and percussion of the sinus areas. Laboratory testing was unremarkable. Chest radiography showed dextrocardia (A); computed tomography (CT) of the face showed mucosal thickening and material with soft tissue density in the paranasal sinuses, mastoid cells and in the middle and external ear cavities (B,C); chest CT showed bronchiectasis and centrilobular nodules (tree-in-bud pattern) (D,E); liver on the left and spleen on the right (F). The diagnosis of Kartagener's syndrome (KS) was made. KS is a subset of primary ciliary dyskinesia, an autosomal recessive condition characterised by bronchiectasis, paranasal sinusitis and situs inversus totalis (SIT). Patients with KS often have multiple episodes of respiratory tract infection and exacerbation of bronchiectasis due to poor mucociliary clearance and some male patients with KS also have sterility due to dyskinesia of the spermatozoa. Clinicians and mainly emergency physicians should be aware of this rare disorder once failure to recognize this syndrome can be potentially hazardous, especially in surgical conditions.

**Figure 1:**
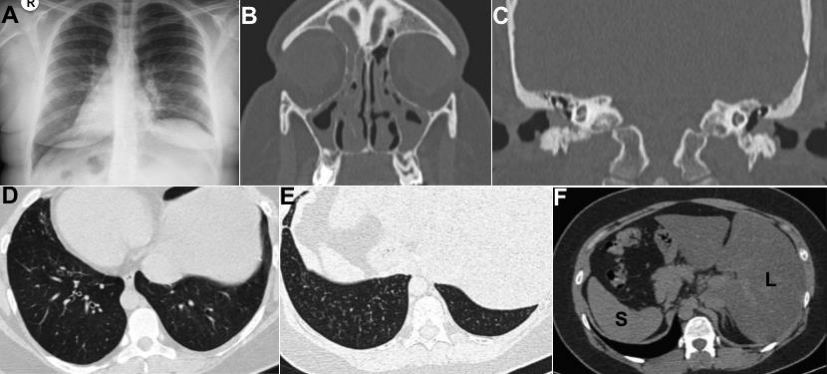
Kartagener’s syndrome: A) chest radiography with dextrocardia; B,C) face CT with mucosal thickening and material with soft tissue density in the paranasal sinuses, mastoid cells and in the middle and external ear cavities; D,E) axial chest CT showing bronchiectasis and centrilobular nodules (tree-in-bud sign); F) axial CT image showing liver (L) on the left and spleen (S) on the right

